# An Investigation of Contrast-Induced Acute Kidney Injury in Patients Undergoing Percutaneous Coronary Intervention: A Cross-Sectional Study From Pakistan

**DOI:** 10.7759/cureus.54726

**Published:** 2024-02-22

**Authors:** Zair Hassan, Usha Kumari, Umaima Wasim, Sanjana Kumari, Nikhil Reddy Daggula, Salim Surani, Hazir Ullah

**Affiliations:** 1 Cardiology, Lady Reading Hospital, Peshawar, PAK; 2 Medicine, Dow University of Health Sciences, Karachi, PAK; 3 Medicine, Ziauddin University, Karachi, PAK; 4 Internal Medicine, Kakatiya Medical College, Warangal, IND; 5 Anesthesiology, Mayo Clinic, Rochester, USA; 6 Medicine, Texas A&M University, College Station, USA; 7 Medicine, University of North Texas, Dallas, USA; 8 Internal Medicine, Pulmonary Associates, Corpus Christi, USA; 9 Clinical Medicine, University of Houston, Houston, USA; 10 Nephrology, Jinnah Teaching Hospital, Peshawar, PAK

**Keywords:** percutaneous coronary intervention complications, contrast-induced kidney injury, contrast-related injury, chronic kidney disease, coronary artery disease, interventional cardiology, coronary angiography, acute kidney injury, medical imaging, nephrotoxicity

## Abstract

Background and objective

Contrast-induced acute kidney injury (CIAKI) is a complication observed among individuals undergoing primary percutaneous coronary intervention (PCI) and is associated with high morbidity and mortality rates. It is characterized by an elevation in serum creatinine (SCr) levels >0.5 mg/dl or a 50% relative increase in SCr from the baseline value following exposure to contrast within a 48- to 72-hour timeframe, in the absence of any alternative causes for acute kidney injury (AKI). This study aimed to assess the incidence of CIAKI in patients following PCI.

Methods

This prospective study was conducted from July to December 2022, after obtaining ethical approval from the institutional ethics committee (reference no: 147/LRH/MTI). A total of 159 consecutive patients who met the selection criteria were enrolled. A detailed patient and family history was obtained, and a thorough physical examination was conducted. Baseline tests, including SCr, were performed, with SCr repeated 72 hours post-PCI. All investigations were performed in the affiliated hospital's main laboratory and conducted by the same biochemist.

Results

The study included 159 patients presenting with myocardial infarction, angina pectoris, or ischemic features on EKG, exercise tolerance test (ETT), or echocardiogram and underwent PCI. The patients had a mean age of 51 ± 9 years, baseline SCr of 0.77 ± 0.41 mg/dl, SCr 72 hours post-procedure of 0.83 ± 0.41 mg/dl, and an average contrast volume of 128.6 ± 63 ml; 87 (55%) patients were male, and 72 (45%) were female. CIAKI was observed in 15 (9.4%) patients. Hypertension and diabetes mellitus were the most prevalent comorbidities. Male gender, diabetes mellitus, and hypertension had a clinically significant association with the development of CIAKI (p<0.05). ST-elevation myocardial infarction (STEMI) was the predominant clinical presentation in 81 (50.9%) cases.

Conclusions

This study examines the frequency, risk factors, and associations of CIAKI following PCI at a tertiary care hospital in a low-middle-income country. We believe our findings provide future directions for identifying and minimizing the risk of CIAKI in this patient population.

## Introduction

Contrast-induced acute kidney injury (CIAKI) is defined as an acute decline in renal function following the intravenous administration of iodinated contrast medium and occurring within 48-72 hours, in the absence of alternative causes for acute kidney impairment [1]. Following the guidelines outlined by Kidney Disease Improving Global Outcomes (KDIGO), the updated definition incorporates "an absolute increase in serum creatine (SCr) of ≥0.3 mg/dl or a relative increase of 50%, typically detected within a 48-hour timeframe" [2]. It represents a significant complication post-PCI and is the third most common cause of hospital-acquired renal failure, following renal artery hypoperfusion and drug-induced nephrotoxicity. It is linked with increased in-hospital mortality and morbidity characterized by dialysis requirements, prolonged hospital stays, and higher costs [2,3]. In a study by Wu et al., the incidence of CIAKI was found to be 9%, with 0.5% of patients requiring renal replacement therapy. Specifically related to coronary angiography, CIAKI occurred at a rate of 9.90%, and dialysis was needed in 0.59% of cases [4]. The likelihood of CIAKI can vary depending on the treatment setting. Studies have shown a higher occurrence of CIAKI following emergency PCI compared to elective PCI [5]. As indicated in another study, the incidence of emergent PCI compared to elective PCI was 10.5% vs. 3.7% (p<0.001) [6].

Although the exact pathophysiological mechanism of CIAKI is not fully elucidated, available evidence indicates a possible connection with nephrotoxicity, inflammatory activation, oxidative stress, the production of reactive oxygen species, and ischemia in the renal medulla, among various other factors [7]. The risk of harm is heightened in the presence of factors such as preexisting renal insufficiency, diabetes mellitus, dehydration, and congestive heart failure [8]. Identifying high-risk groups promptly is crucial, particularly for the proactive prevention and treatment of CIAKI.

Recent years have seen the establishment of risk scores to assess the likelihood of developing CIAKI after PCI. The Mehran score, or the updated Mehran 2 risk score, is considered the gold standard for accurately predicting CIAKI risk. This scoring system includes the following eight clinical factors: age, coronary artery disease subtype, estimated glomerular filtration rate (eGFR), diabetes mellitus, congestive heart failure, hemoglobin, fasting plasma glucose, and left ventricular ejection fraction. However, its practical applicability is limited due to the model's timeliness and its exclusion of patients with acute myocardial infarction [9].

Furthermore, the intra-arterial method of administration contrast medium is associated with a greater risk of CIAKI than the intravenous approach. The specific process is uncertain; however, it is widely assumed that undiluted contrast medium reaching the nephrons has direct harmful consequences [10]. The volume of contrast medium provided and the risk of CIAKI are directly connected. Ozturk et al. observed a 12-28% increase in risk for every 100 ml of contrast administered. They concluded that individuals undergoing complicated PCI had the highest incidence and clinical impact of CIAKI [11]. In Japan, Inohara et al. reported an alarming statistic: 10.5% of patients developed CIAKI following PCI [12].

In this study, we aim to quantify the burden of CIAKI in patients undergoing PCI at our hospital. While several studies on this topic have been conducted worldwide, there is scarce data from our local settings. This study aims to calculate the rate of CIAKI in patients with different baseline characteristics, clinical presentation, and contrast volume. The results could guide us better in terms of patient selection, intraprocedural measures (e.g., cautious use of contrast volume administered), and preventive treatment plans to reduce CIAKI incidence in post-PCI patients, thereby alleviating physical and financial burdens on patients and the healthcare system.

## Materials and methods

This descriptive study was conducted at Lady Reading Hospital Medical Teaching Institute, a tertiary care hospital in Pakistan, from July 2022 to December 2022. Employing a non-probability consecutive sampling technique, the investigation aimed to delve into the incidence of CIAKI following PCI. Ethical approval was obtained from the institutional ethics committee (reference no: 147/LRH/MTI).

The sample size was computed by using the World Health Organization (WHO) calculator, guided by a predefined frequency of 7.1% for CIAKI post-PCI, a margin of error set at 5%, and a confidence interval of 95% [8]. A total of 159 consecutive patients who met the selection criteria were enrolled. Patients presenting with myocardial infarction, angina pectoris, or features of ischemia on EKG, exercise tolerance test (ETT), or echocardiogram requiring PCI were included. The patients were aged between 30 and 70 years old. The reason for selecting this age bracket was to avoid outliers, as the majority of patients who undergo PCI in Pakistan are aged above 40 years, with average ages for males and females of 51.9 and 51.3 years, respectively [13]. Individuals experiencing sepsis, renal artery stenosis, chronic kidney disease (CKD), disseminated intravascular coagulation, shock, or hematuria, or very elderly individuals were excluded. This exclusion criterion aimed to mitigate potential confounding factors that could introduce bias and impact study outcomes.

Informed consent was diligently obtained from patients or their guardians before their participation, ensuring that they had a comprehensive understanding of the study's objectives and procedures. Baseline investigations were initiated, with SCr levels reassessed 72 hours post-PCI. To ensure consistency, all investigations were conducted by the same biochemist. The tests were performed on the Cobas Pro c 503 machine (F. Hoffmann-La Roche AG, Basel, Switzerland). In the absence of routine urine output measurement in noncritically ill patients, only SCr-based criteria were used to define acute kidney injury (AKI) in this study. As per universal guidelines, CIAKI was diagnosed based on an increase of ≥50% in SCr or a level of ≥0.3 mg/dl, typically detected within a 72-hour timeframe following contrast use in the absence of other risk factors. The data were meticulously recorded in an anonymized proforma, minimizing potential biases. Subsequently, all data were entered into the statistical software SPSS Statistics 20.0 (IBM Corp., Armonk, NY), facilitating a robust descriptive analysis. Quantitative variables, such as age, were elucidated in terms of mean and standard deviation (SD). Categorical variables, including gender, obesity, hypertension, diabetes mellitus, congestive heart failure, smoking status, and CIAKI, were expounded in frequencies and percentages. To assess potential effect modification, variables such as congestive heart failure, smoking status, stable or unstable angina, and myocardial infarction type were scrutinized post-stratification. The Chi-square test was then employed, with statistical significance set at a p-value of ≤0.05.

## Results

**Figure 1:**
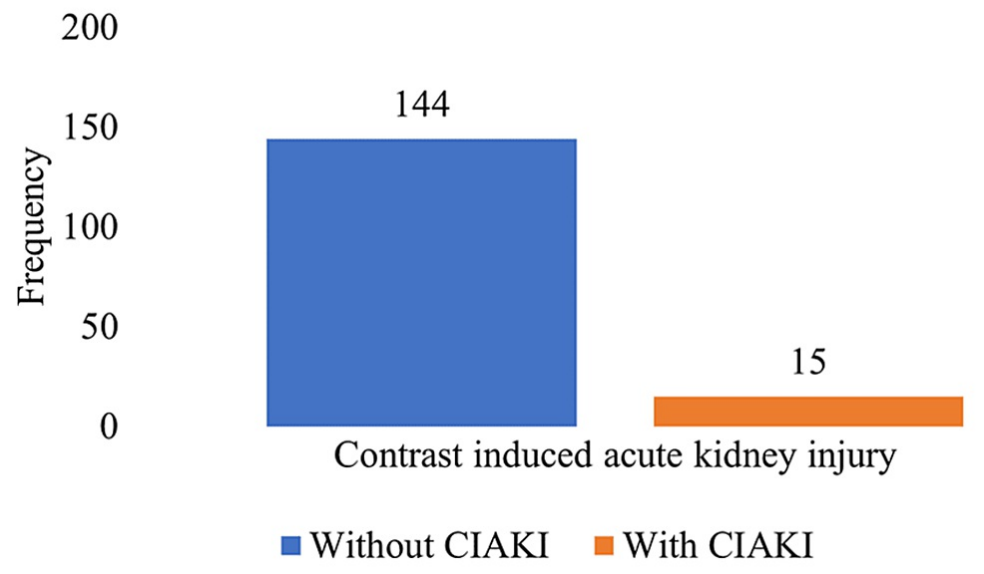
Frequency of CIAKI after percutaneous coronary intervention (N=159) CIAKI: contrast-induced acute kidney injury

A total of 159 patients presenting with myocardial infarction, angina pectoris, or features of ischemia on EKG, ETT, or echocardiogram and underwent PCI were included. The average age of the patients was 51±9 years (range: 30-69 years). The mean initial baseline SCr was 0.77 ± 0.41 mg/dl (range: 0.11-1.45 mg/dl). The mean of 72 hours post-procedure SCr was 0.83 ± 0.41 mg/dl (range: 0.13-1.53 mg/dl). The average volume of contrast administered was 128 ± 63 ml (range: 30-249 ml). The frequency of CIAKI is represented in Figure 1.

In the cohort, there were 87 (55%) males and 72 (45%) females. Hypertension was the most common comorbidity (n=86, 54%), followed by diabetes mellitus (n=55, 34.6%), smoking (n=42, 26.4%), and obesity (n=37, 23%). The most common clinical presentation was ST-elevation myocardial infarction (STEMI), seen in 81 patients (50.9%), followed by non-ST-elevation myocardial infarction (NSTEMI), unstable angina, and stable angina with a frequency of 31 (19.4%), 30 (18.8%), and 17 (10.6%), respectively. A preexisting risk factor in patients with CIAKI after PCI showed a statistically significant association with male gender, diabetes mellitus, and hypertension (p<0.05), as shown in Table 1. A comparison of clinical presentations with CIAKI after PCI revealed that STEMI and unstable angina were associated with CIAKI (p<0.05). However, no statistically significant association was found between CIAKI after PCI and stable angina and NSTEMI.

**Table 1:** Comparison of preexisting risk factors in patients with CIAKI after percutaneous coronary intervention (n=159) *Statistically significant (p<0.05) association CIAKI: contrast-induced acute kidney injury

Risk factors	Frequency (%)	
	Yes	No
Male gender*	12 (7.6%)	147 (92.4%)
Age >50 years	7 (4.4%)	152 (95.6%)
Diabetes mellitus*	11 (6.9%)	148 (93.1%)
Hypertension*	12 (7.6%)	147 (92.4%)
Smoking	4 (2.6%)	155 (97.4%)
Obesity	3 (1.9%)	156 (98.1%)

## Discussion

While exposure to contrast media is a significant risk factor for AKI in patients undergoing PCI, recent studies indicate that other factors such as advanced age, diabetes mellitus, and CKD also play a crucial role in causing renal dysfunction [14]. Diabetes intensifies the vulnerability to preexisting CKD. In diabetes, higher glucose levels lead to more oxidative stress and reactive oxygen species (ROS). This causes an increase in vasoactive substances like endothelin and angiotensin II, leading to issues like reduced blood flow, disruptions in kidney microcirculation, and increased constriction of renal blood vessels. Ischemia, in turn, can trigger an escalation in oxygen-free radicals and ROS formation, establishing a detrimental cycle [7].

Elderly patients are at an increased risk of developing CIAKI due to a decrease in nephrons, inadequate renal functional reserve, and the presence of comorbidities. Their prognosis is poorer compared to the general population. One study estimates the incidence of CIAKI to be over 2% in the general population, but in high-risk groups with factors predisposing them to kidney disease, the incidence rises significantly to 20-30% [15]. A large contemporary study reported that pre-procedure characteristics such as STEMI and CKD were strong predictors of AKI, and they led to a 2-28-fold increase in the odds of developing CIAKI [16]. The outcomes reported in our study appear to be comparatively lower than those in the literature, which could be attributed to the predominant inclusion of patients with acute coronary syndrome in the study sample as opposed to individuals with stable ischemic heart disease. Patients undergoing primary PCI have a higher risk of CIAKI compared to those with elective PCI, mainly due to unstable blood flow and a lack of effective measures to prevent kidney issues [17]. We also observed a higher incidence of CIAKI in older individuals, with 18% of patients aged 65 or older experiencing this complication. This underscores the clinical significance of early and accurate risk assessment and the implementation of tailored preventive measures.

This rise in SCr following kidney damage is often delayed [18]. In a recent study, peripheral blood mononuclear cells (PBMCs) were utilized to investigate how contrast media influences oxidative stress, inflammation, mitochondrial function, and cell death. The objective was to uncover connections between these factors and the occurrence of CIAKI. This study reported that the mitochondrial dysfunction in PBMCs occurs before the rise in SCr, indicating its potential utility as an early biomarker for CIAKI [19]. A study by Qiu et al. found high-sensitivity C-reactive protein (hs-CRP) to be an independent risk factor for CIAKI. They also suggested that patients with CIAKI exhibit higher levels of hs-CRP than patients without CIAKI. However, the risk-scoring model (Mehran's) did not include the mediators related to inflammation [7].

Studies have suggested a significant association between elevated serum levels of gamma-glutamyl transferase (GGT) and the burden of CIAKI, implying that GGT levels could serve as a valuable predictor for the onset of AKI following cardiac catheterization. GGT, an enzyme located on the plasma membrane, transfers glutathione - a crucial antioxidant - and serves as a long-term indicator of renal function and microalbuminuria. Serum GGT serves as an important predictor and its incorporation into the scoring system as Mehran risk score could enhance the prediction of CIAKI [20]. While pre-intervention measures for CIAKI during PCI have been thoroughly studied, less emphasis has been placed on procedural strategies to mitigate the risk. Factors such as adequate hydration, avoiding nephrotoxic medications, judicious use of contrast, and reducing contrast volume via strategies or techniques like intravascular ultrasound or non-contrast-based optical coherence tomography or ultra-low-contrast-volume PCI protocol can be of importance in preventing CIAKI [21].

An examination revealed that employing intravascular ultrasound during PCI was linked to reduced rates of cardiovascular mortality, myocardial infarction, target lesion revascularization, and stent thrombosis when compared to relying solely on coronary angiography. This approach is both feasible and safe for patients with CKD. Even when dealing with intricate lesion morphologies, the procedure can be carried out without the use of contrast, thereby minimizing the risk of long-term renal and cardiovascular complications [22]. Another randomized clinical trial employing personalized intravenous fluid plans adjusted based on measurements of left ventricular end-diastolic pressure and central venous pressure has also documented decreases in the occurrence of AKI following coronary procedures in patients [23]. The potential effect of coenzyme Q10 administration as an adjuvant to saline hydration for the prevention of CIAKI in patients with STEMI undergoing primary PCI has also been proven beneficial in a recent study conducted by Ahmadi Moghaddam et al. [24].

Probucol, a robust antioxidant medication with anti-apoptotic characteristics, may offer kidney protection against CIAKI. One study has demonstrated that the combination of probucol and hydration has effectively decreased the occurrence of CI-AKI in individuals with coronary heart disease undergoing coronary angiography or PCI [25]. Despite the existing varied strategies such as maintaining adequate volume expansion in the peri-procedure period, minimizing the volume of contrast media, and avoiding the use of nephrotoxic medications, there are still no definitive guidelines regarding the most effective intervention to prevent or reduce this complication [26]. CIAKI following PCI is associated with significantly longer hospital stays, higher admission costs, and long-term treatment. Excessive costs place a heavy burden on hospitals, and the additional expenses for payers are not clearly defined. Gaining insights into these ongoing expenditures could provide clarity for decision-making and potentially inform the implementation of future policies [26].

This study has a few limitations. The impact estimates in the model are derived from interventional and prospective observational studies, and they lack extended follow-up to assess the long-term outcomes and consequences for patients following the intervention. The study was conducted among a small population in Pakistan and hence its findings could not be generalized on a broader scale. The impact of CIAKI on the young population could not be analyzed as the overall incidence of CAD requiring PCI in this demographic is much less in our population.

## Conclusions

Contrast-induced acute renal damage is a common complication in cardiac catheterization patients and is associated with very poor clinical outcomes, including high mortality rates. In our study, 9.4% of patients were observed to have CIAKI following PCI, without any other identifiable causes for acute kidney damage. We found significant associations with male gender, diabetes, and hypertension. This study underscores the importance of considering these factors in the assessment and management of patients undergoing procedures involving contrast media. An effective preventative method relies on limiting contrast delivery and intravenous fluid hydration. Further research into novel markers of kidney damage may enable early identification and prompt management of CIAKI.
